# Community Health Workers and Mobile Technology: A Systematic Review of the Literature

**DOI:** 10.1371/journal.pone.0065772

**Published:** 2013-06-12

**Authors:** Rebecca Braun, Caricia Catalani, Julian Wimbush, Dennis Israelski

**Affiliations:** 1 Bixby Center for Population, Health and Sustainability, University of California, Berkeley, California, United States of America; 2 Innovative Support to Emergencies, Diseases and Disasters (InSTEDD), Sunnyvale, California, United States of America; 3 University of California, Berkeley, California, United States of America; 4 Stanford University School of Medicine, Palo Alto, California, United States of America; The University of Auckland, New Zealand

## Abstract

**Introduction:**

In low-resource settings, community health workers are frontline providers who shoulder the health service delivery burden. Increasingly, mobile technologies are developed, tested, and deployed with community health workers to facilitate tasks and improve outcomes. We reviewed the evidence for the use of mobile technology by community health workers to identify opportunities and challenges for strengthening health systems in resource-constrained settings.

**Methods:**

We conducted a systematic review of peer-reviewed literature from health, medical, social science, and engineering databases, using PRISMA guidelines. We identified a total of 25 unique full-text research articles on community health workers and their use of mobile technology for the delivery of health services.

**Results:**

Community health workers have used mobile tools to advance a broad range of health aims throughout the globe, particularly maternal and child health, HIV/AIDS, and sexual and reproductive health. Most commonly, community health workers use mobile technology to collect field-based health data, receive alerts and reminders, facilitate health education sessions, and conduct person-to-person communication. Programmatic efforts to strengthen health service delivery focus on improving adherence to standards and guidelines, community education and training, and programmatic leadership and management practices. Those studies that evaluated program outcomes provided some evidence that mobile tools help community health workers to improve the quality of care provided, efficiency of services, and capacity for program monitoring.

**Discussion:**

Evidence suggests mobile technology presents promising opportunities to improve the range and quality of services provided by community health workers. Small-scale efforts, pilot projects, and preliminary descriptive studies are increasing, and there is a trend toward using feasible and acceptable interventions that lead to positive program outcomes through operational improvements and rigorous study designs. Programmatic and scientific gaps will need to be addressed by global leaders as they advance the use and assessment of mobile technology tools for community health workers.

## Introduction

Nearly all countries are challenged by shortages of health workers [1]. For the world’s poorest countries, the scarcity of human resources is a crisis fueled by the migration of qualified health workers to richer countries, inadequate investment in national health systems, and devastation of major epidemics such as HIV/AIDS, tuberculosis, and malaria [2]. Meanwhile, global health donors and advocates, ministries of health, and local leaders have higher demands and more ambitious hopes for health systems in resource-constrained settings than ever before [3]. To achieve the Millennium Development Goals and other global aims, several health policy organizations are leading campaigns to further engage community health workers (CHWs) through task shifting [4], decentralized distribution of health services [5], and other mechanisms to galvanize local communities to provide health services.

In low resource settings, CHWs build bridges between formal health systems and communities, working to improve the relevance, acceptability, and accessibility of health services [6]. CHWs serve many functions, including conducting home visits, assessment and treatment of disease, data collection, education and counseling and referrals for further care. By directly visiting households, CHWs can increase access to care for groups who are particularly difficult to reach, such as secluded women, the extremely poor, or the lowest classes of society. With their links to the health system, CHWs can also offer an entry point for and at times directly provide health services, such as contraceptive methods, home-based care for people living with AIDS, directly observed therapy of tuberculosis, and community-integrated management of childhood illnesses [7].

Proponents argue engaging CHWs expands the pool of human resources for health, improves the productivity of health systems, and lowers the cost of providing services by shifting tasks from highly trained physicians and nurses to less specialized community members [2], [3], [5]. The evidence for the effectiveness of CHW programs varies. Much research demonstrates that health interventions integrating CHWs can lead to positive behavior changes and lower morbidity and mortality rates, while moving services closer to the communities where they are actually needed. Further, some CHW programs report equally high quality of care at lower cost when compared to traditional approaches [8]. Among other programs, however, data show that quality of health services delivered by CHWs may be compromised without proper investments in supportive organizational policies, adequate supervision and mentorship, quality trainings, and sufficient program resources [9].

Increasingly, new mobile information technologies are being developed, tested, and piloted with CHWs. The use of mobile technology by CHWs to improve healthcare services has intuitive appeal. mHealth tools enable CHWs to provide health services far from the clinical setting, in remote areas, and among hard to reach communities. Under this decentralized approach to service provision, health care can become more accessible to patients due to reduced time and expense of travel [10] and due to the ability to seek out patients who are the targets of stigma and discrimination [11]. These tools may help CHWs overcome many of the barriers they face in the field, including balancing multiple priorities, lacking appropriate tools to provide services and collect data, and limited access to training and supervision. As CHWs are generally the most frequent connectors of communities to formal health systems, the use of mobile tools to enhance CHW performance warrants further study. The explosive innovation in technology has led to a proliferation of pilot initiatives and heterogeneity of research designs and outcome measures. Thus we systematically reviewed the literature to provide a critical assessment of the evidence to date on the use of mobile technology to help improve the services delivered by CHWs and the health of the communities they serve.

## Methods

### Search Strategy

We conducted a systematic review of literature published in English between January 1, 2000 and June 30, 2012 using the PRISMA statement as guidance [12]. First, to capture the multidisciplinary evidence of this field, we searched in the following medical, public health, engineering, and global development databases: Pubmed/Medline, CAB Global Health, Web of Science, and INSPEC. Second, we searched the following targeted institutional databases: WHO publication database, Health UnBound (HUB) Content Library, and Royal Tropical Institute resource database. Third, we searched citations within the first round of articles to identify additional relevant literature.

### Inclusion and Exclusion Criteria

**Figure 1:**
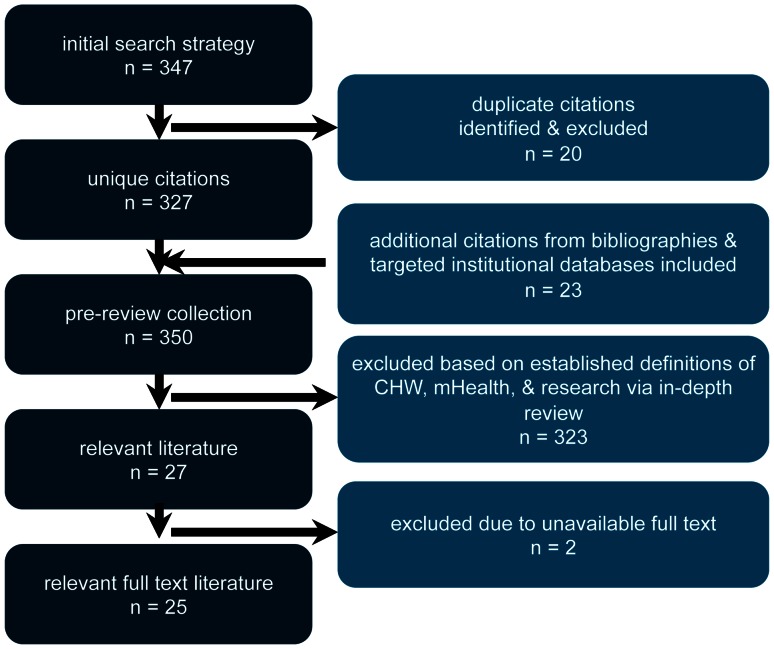
Literature Search Strategy.

We searched for articles referring to both mHealth and CHWs in the title or abstract. To refer to mHealth, articles either had to include the term ‘mHealth’, or include both the term ‘health’ and one of the following search terms: handheld computer, mobile phone, cellular phone, mobile device, patient monitoring device, mobile telemedicine, MP3 player, mobile operating system technology, 3G, SMS, text message, IVR, interactive voice response, GPS or global positioning system. To refer to CHWs, articles had to include one of the following search terms: community health worker, frontline health worker, midwife, outreach worker, community health education worker, lay health worker, promotora, village health worker, volunteer health worker, community health distributor, community health surveyor, community health assistant, community health promoter, community health agent, rural health auxiliaries, traditional birth attendant, or health promoter. This initial search strategy yielded 347 articles. Duplicate citations across databases were identified and excluded, and relevant citations from bibliographies and targeted institutional databases were identified and included. We then conducted an in-depth review of the full text of this subsequent set of articles. We included those articles that specifically focused on the use of mHealth technology by CHWs, and excluded those articles that did not meet our definition of research, such as commentaries, policy briefs, systematic reviews and other summary-type articles. After further excluding articles without available full text, the final collection for review included 25 articles (Figure 1).

### Data Collection

To assess the literature, two analysts systematically coded articles to describe the following topics: study design, methods, unit of analysis, number of participants, findings, purpose of technology, mHealth platforms/applications, theoretical framework, location, population served, health issues addressed, information architecture, open source tools, interoperability, engagement/participation of CHWs, strategies for organizational strengthening, outcomes for organizational performance, and conclusions. As a team, analysts defined and discussed key topics listed above, compiled findings by populating tables according to key topics, and discussed and reconciled any differences.

### Data Synthesis & Analysis

To provide a conceptual framework for the review and enable comparison across projects, we used the World Bank’s *Improving the Delivery of Health Services: A Guide to Choosing Strategies* [13], commonly used in global health and development to guide programmatic and policy decisions. We used recommended outcomes for organizational performance (quality, efficiency, utilization, access, learning, and sustainability) and strategies for improving organizational performance (standards and guidelines, organizational design, education and training, process improvement and technology and tool development, incentives, organizational culture, and leadership and management) as a means of categorizing, synthesizing, and assessing each programs’ reported processes and outcomes. A complete summary of all articles in the systematic review is available in the supporting information section (Table S1).

## Results

### Scope of Research

The 25 articles reviewed reported on 28 unique studies, as one article described four distinct studies. Most articles reported on projects in developing countries particularly Africa, with several focused on Asia, and a few in Latin America. There were more programs in rural than urban areas, though a few studies operated in both. A broad range of health issues were addressed; the most common included the interrelated set of issues around sexual, reproductive, maternal and child health, of which more than half focused specifically on HIV/AIDS. Other key issues included tuberculosis and malaria. mHealth technology was most commonly used for data collection, decision support, alerts and reminders, and information on demand, facilitating CHW activities associated with field-based research and direct provision of medical care (Table 1).

**Table 1:** Overview of Scope of Research.

Category	Subcategory	Result (n)	Result (%)
Rural/Urban	Rural	15	54%
	Urban	9	32%
Region	Africa	19	68%
	Asia	5	18%
	Latin America	3	11%
Health Issue Addressed	Sexual, Reproductive, Maternal & Child Health	11	39%
	HIV/AIDS	10	36%
	Tuberculosis	3	11%
	Malaria	2	7%
Purpose of Technology	Data Collection	16	57%
	Decision Support	6	21%
	Alerts & Reminders	6	21%
	Information on Demand	5	18%

### Research Designs and Methods

Our review revealed that research and evaluation related to CHWs and mHealth had substantial variation in design and methods (Table 2). More of the studies were quantitative than qualitative, while several articles employed both methodologies. Ten studies were non-experimental, typically including a descriptive assessment of an mHealth technology or program without any assessment of outcomes or impacts. Ten studies were quasi-experimental, designed with a control group, frequently comparing the computer-based experimental intervention to a traditional paper-based control intervention–but without randomization of participants.

**Table 2:** Overview of Research Design & Methods.

Category	Subcategory	Result (n)	Result (%)
Methods	Quantitative	15	54%
	Qualitative	7	25%
	Both	6	21%
Design	Non-Experimental	10	36%
	Quasi-Experimental	10	36%
	Experimental	8	29%

Eight studies were experimental, designed with randomized selection of control and intervention groups. All of these studies were published after 2010. These include a description of a cluster-randomized controlled trial with CHWs to evaluate effects of a health data collection and decision support system on health outcomes of mothers living with HIV [14], a cluster randomized controlled trial to evaluate effects of alerts and reminders on CHWs adherence to malaria treatment guidelines [15], and a randomized crossover study to evaluate use of mobile multimedia for simulated patient interactions to enhance performance of CHWs [16]. These also include a mixed methods evaluation of a cluster-randomized trial to evaluate use of mHealth tools by peer health workers providing services to HIV infected clients [17], and a series of randomized-control studies investigating use of automated short message services (SMS) to improve CHW performance when delivering general health services [18].

Overall, the literature revealed a general tendency toward increased use of experimental research designs. Standards for reporting results differed between articles, however, and often reflected differences in journal requirements and style standards between disciplines from computer engineering to social science to medicine.

### Strategies for Strengthening Health Organizations & Systems

There were four key strategies to improving the delivery of health services by CHWs through use of mHealth tools: (1) process improvement and technology development, (2) standards and guidelines, (3) education and training, and (4) leadership and management. First, we found that process improvement through technology development was inherent to almost all studies (n = 27, 96%) and the literature supported the aim of developing interventions that are more efficient and less susceptible to human error through the creation of better tools and workflow systems. The most typical task for CHWs is the collection of field-based data, whether for client care, program monitoring, or health research. According to the literature, when equipped with mobile devices, CHWs became capable collectors of complete, high quality, and timely data from the field. More specifically, as compared to paper-based data collection, data collected by CHWs using mHealth tools had fewer errors [19] and less data loss [20]. Further, these mobile tools can enable real-time quality review and analysis for decision-making [11], as well as rapid response to cited health issues [20].

Second, a third of studies (n = 9, 32%) used mHealth tools to ensure CHW compliance to standards and guidelines for health services in the field, most prominently through decision support, and alert and reminder tools. For example, a study using text messaging to improve outpatient malaria care in 107 government health facilities in Kenya led to improvements in drug management both immediately after the intervention, and six months post-intervention [21]. Qualitative interviews demonstrated high levels of satisfaction with all aspects of the intervention.

Third, 28% (n = 8) of studies described the use of mHealth tools to support education and training for CHWs. Studies assessed the use of mHealth tools to reach geographically dispersed CHWs with accurate and timely clinical information, shared through multimedia formats [16], [22]. mHealth tools were also used to facilitate the creation of professional networks, both among CHWs and between CHWs and their supervisors, providing real-time advice, information, and support for frontline health workers. In the Aceh-Behar midwives study in Indonesia, the use of mobile phones was positively associated with access to institutional and peer information resources, which was in turn positively associated with an increase in knowledge about best practices for providing obstetric care [23]. Similarly, the k4Health project in Malawi recently introduced an SMS text-messaging network to improve the exchange and use of reproductive health and HIV/AIDS information among CHWs. After an 18-month pilot period, the authors found that CHWs who used the text-message network were more likely to contact supervisors for clinical support from the field. The authors argue that timely exchange of information led to improved quality of care, particularly in cases of obstetric emergency [22].

Fourth, a quarter (n = 7, 25%) of the studies suggest that mHealth tools can be used to facilitate better practices in leadership and management, particularly in terms of providing remote supervision to CHWs. A salient example, the Tanzania CommCare project, used an automated text-message system to remotely monitor real-time job performance of midwives and to provide workers with alerts and reminders to their mobile phone about past-due patient visits [24]. Compared to a group of midwives who did not receive alerts and reminders, midwives who received these messages improved the number of timely visits to expectant mothers. In further studies, researchers found the comparative effect of adding a phone call from supervisors to alert midwives about missed and late visits was associated with an 86% reduction in the number of days that CHWs were overdue in visiting their clients. Finally, in-depth interviews with CHWs and supervisors revealed high rates of acceptability, use, and satisfaction with the alert and reminder system [18]. The evidence suggests that mHealth tools, in hands of CHWs and committed supervisors, can facilitate real time supervision of teams of health workers that are distributed widely in geography.

Although there are several examples from the literature of tools used to improve supervision of CHWs, there are very few instances of interventions to improve CHW leadership. In one of the few articles to explore the use of mHealth tools to enhance CHW leadership, researchers spent six months working with frontline health workers and hospital staff to design a pilot tuberculosis program in Malawi. Taking advantage of the leadership and expertise of frontline workers, the team designed and developed a system for patient adherence reporting, appointment reminders, and physician queries [10]. Similarly, in a small pilot study of a maternal health project in Tanzania, researchers met with five CHWs weekly over four months to collaboratively design and develop a tool for use in delivery maternal and child health services They also conducted field-observations of CHWs providing household visits for prenatal care. Authors concluded that their design process resulted in building a tool that was responsive to CHW needs facilitating locally driven innovation and ownership [24].

### Health Organization & System Outcomes

Several studies provided evidence that mHealth tools can improve outcomes related to CHW performance, most principally: quality of care, efficiency of services, CHW learning, and utilization of services. The outcome most frequently measured among studies (n = 20, 71%) was improvements in quality of care when CHWs use mobile tools, most commonly measured in terms of compliance to accepted standards of care. For example, a simulated experimental study used mobile multimedia devices to support point-of-care clinical decisions by CHWs in Colombia and found that CHWs had significantly fewer errors and better compliance with care protocols over a range of clinical care situations. The authors argued that although CHWs are the backbone of health care delivery in developing countries, they too often have little formal education and training, and so devices that use a combination of text, audio, images, and video can be used to improve their ability to provide quality community based care [16].

Use of mHealth tools was frequently measured with regard to general improvements in efficiency. There was no consensus, however, as to the definition and best measures for CHW efficiency across the literature reviewed. In one study, improvements in efficiency were measured in terms of savings in cost and time and increased work capacity. At the end of the pilot study in Malawi, mobile phones with FrontlineSMS used by 75 CHWs over the course of six months saved approximately 2,048 fewer hours of worker time and $2,750 less in fuel and operational costs, which in turn led to the ability to double the number of clients served by CHWs [10].

Learning as an outcome was cited in a third of the articles (n = 9, 32%). In the Aceh-Behar study, mobile phones use was positively associated with higher self-efficacy (.25, p<.001) among CHWs, as measured through a series of items regarding confidence about their abilities. Higher self-efficacy was positively associated (.16, p<.05) with health knowledge of maternal health practices in the areas of family planning practices, prenatal, and child delivery processes [23]. Further, the traditional birth attendants in the study explained that use of mobile phones enhanced their ability to independently meet the clients’ health care needs through more timely access to relevant information [25].

## Discussion

Overall, this systematic review revealed that the number and types of mHealth-focused CHW project evaluations have grown during the past twelve years. There has been a positive trend toward increased use of experimental designs and methods, particularly in the past three years. The findings of this review demonstrate that CHWs are using mHealth tools with increasing effectiveness to improve the delivery of maternal and child health, HIV and other sexual and reproductive health services, and other general health services in the developing world, mainly in Africa. Moreover, given the great potential for mHealth tools to be incorporated into customary workflows of CHWs, it is expected that the use of mobile technologies can be broadened to support and empower CHWs in their role as a bridge between formal health systems and communities.

Social, policy, and technical challenges remain. The majority of studies were pilots and provided little or no information about the effectiveness or impact of the use of mobile technologies when included in large-scale implementations of well-architected electronic health strategies. More than just multiplying the size of pilot programs, large scale or national implementations of CHW mHealth programs may require policies to support the integration of CHWs into national strategies for health system strengthening [26], more attention to adoption of shared technical standards, consistent use of open standards and open source tools, and a promotion of interoperable information systems that can comprehensively address the full spectrum of needs around health information exchange [27]. A recently published article on the RapidSMS system to monitor pregnancy and reduce MCH deaths in Rwanda demonstrates the potential for successful scale-up of mHealth tools used by CHWs when there is strong government ownership of the process [28].

The literature demonstrates the usefulness of mHealth tools to facilitate process improvements and compliance with standards and guidelines. Moreover, per Berman et al [13], significant potential remains in the areas of education and training, organizational culture and design, leadership and management. Several CHW advocates argue that the most successful projects engage CHWs as leaders and experts [19]; minimal evidence exists on engaging CHWs as leaders, however, and scarcely any articles mentioned evolving organizational cultures to improve performance outcomes.

Mobile technologies show great potential to enhance CHWs’ opportunity to participate in design, implementation and evaluation processes, yet the literature reveals that this user centric design is more the exception than the rule. mHealth initiatives more commonly result from a top-down approach to organizational change that aims to ensure CHW adherence to existing guidelines, policies and procedures but with minimal support from home institutions, supervisors, or other CHWs. Thus per “Conway’s Law” [29], the deployment of mHealth tools to support CHWs are likely to mirror the communication structures of the organizations that design them. Greater than the challenges related to the technology design and computer engineering are the policy barriers to institutionalizing CHWs as an accepted part of the health system.

Although CHWs are increasingly using mHealth tools to enhance delivery of community-based healthcare services and to access continued learning in the field, there remains a need for more rigorous measurement of improvements in performance and outcomes, eg. service utilization, access, productivity, quality and sustainability. Cost effectiveness analysis would be helpful for program staff and policy makers, as they must decide the value of national implementations and make operational decisions about using mobile technology to deliver program targets. The existing literature provides little or no evidence around calculating costs, including both initial investments and maintenance over time, and measuring efficiency or benefits.

### Limitations

Our review is limited by the scope of our literature search, which included articles in English collected through scholarly and organizational databases. As most projects are focused on deployment of tools to enhance service delivery rather than scientific interests, it is likely many mHealth projects are not reported, presented at prominent conferences or published in peer-reviewed journals. Although overviews of the field indicate that CHWs are very commonly engaged in mHealth projects, our collection of only 25 articles is not likely to represent the full range of projects being implemented. Among those that are reported, negative results are less likely to be published. As such, this review may be biased toward more affirmative results and substantial impacts. Further, due to the heterogeneity of methods, units of analysis, and design approaches in this newly evolving field of research, a meta-analysis was not feasible or meaningful.

### Conclusions

There is a growing body of evidence for the use of mHealth tools to improve effectiveness of CHWs in resource-constrained settings. Use of mobile technology can potentially enhance the capacity of CHWs to take on new and challenging tasks, particularly collecting complete, timely and accurate health data for field-based research and providing health care services in the field with fewer errors and higher adherence to protocols. Given the gaps in the literature, future research efforts should include a focus on qualitative analysis, in order to better understand how mHealth tools may be better designed to enable the performance of CHWs globally. Further, a focus on impact evaluation using comparable standards and indicators across studies would accelerate the evidence base needed to inform implementations of systems of care that incorporate mobile technologies. The potential for CHWs to use mobile tools to improve health service delivery in resource limited settings is certainly great; however, a stronger evidence base is necessary to guide global health policy and program implementation.

## Supporting Information

Table S1 **Summary of Systematic Review.**
(PDF)
