# Supplementary material for: Community Health Workers and Mobile Technology: A Systematic Review of the Literature
Source: PLoS One. 2013 Jun 12;8(6):e65772. doi: 10.1371/journal.pone.0065772 (PMC3680423; doi:10.1371/journal.pone.0065772)
Supplement: Table S1 — Summary of Systematic Review. (PDF) [file pone.0065772.s001.pdf]

Summary of Systematic Review

First Author	Pub Year	Article Title	mHealth Tool	mHealth Platform or Application	Region	Health issue addressed by CHW	Purpose of technology	Design			Methods		Taxonomy of Strategies (Berman)							Outcomes of Organization Performance (Berman)					
								Non-experimental	Quasi-experimental	Experimental	Qual	Quant	Standards & Guidelines	Organizational Design	Education & Training	Proc Improvmt, Tech Dvmt	Incentives	Organizational Culture	Leadership & Management	Quality	Efficiency	Utilization	Access	Learning	Sustainability
Andreatta	2011	Using cell phones to collect postpartum hemorrhage outcome data in rural Ghana.	Basic mobile phones	Global System for Mobile Communication (GSM)	Africa	MCH	Health Data Collection	x				x			x	x				x					
Bernabe-Ortiz	2008	Handheld computers for self-administered sensitive data collection: A comparative study in Peru	Personalized Digital Assistant (PDA)	PDA PREVEN	Central/ South America	Sexual & reproductive health	Health Data Collection		x			x				x				x					
Birnbaum	2012	Automated quality control for mobile data collection	Basic mobile phones	CommCare	NA	NA	Data Quality Control		x			x				x									
Chang	2011	Impact of a mHealth intervention for peer health workers on AIDS care in rural Uganda: a mixed methods evaluation of a cluster-randomized trial	Basic mobile phones	Short Message Service (SMS)	Africa	HIV/AIDS	Information on Demand - Health Data Collection - Person-to-Person Communication			x	x	x	x			x		x	x	x				x	
Chib	2010	The Aceh Besar midwives with mobile phones project: Design and evaluation perspectives using the information and communication technologies for healthcare development model	Mobile phones equipped with GRPS technology	Short Message Service (SMS)	Asia	MCH, Sexual & reproductive health	Health Data Collection - Person-to-Person Communication		x		x	x	x			x		x	x	x	x		x	x	
Chib	2008	Midwives and mobiles: using ICTs to improve healthcare in Aceh Besar, Indonesia	Mobile phones equipped with GRPS technology	Short Message Service (SMS)	Asia	MCH, Sexual & reproductive health	Health Data Collection - Person-to-Person Communication		x		x		x			x		x	x	x	x		x	x	
Curioso	2005	Design and Implementation of Cell-PREVEN: A Real-Time Surveillance System for Adverse Events Using Cell Phones in Peru	Basic mobile phones	Cell PREVEN	South America	Sexual & reproductive health, HIV/AIDS	Alerts & Reminders -- Information on Demand -- Health Data Collection -- Electronic Health Record	x				x				x				x				x	
DeRenzi	2012	Improving community health worker performance through automated SMS	Basic mobile phones	CommCare	Africa	MCH and general health	Alerts & Reminders			x		x				x					x	x			x
DeRenzi	2012	Improving community health worker performance through automated SMS	Basic mobile phones	CommCare	Africa	MCH and general health	Alerts & Reminders			x		x				x			x		x	x			x
DeRenzi	2012	Improving community health worker performance through automated SMS	Basic mobile phones	CommCare	Africa	MCH and general health	Alerts & Reminders			x		x				x			x		x	x		x	x
DeRenzi	2012	Improving community health worker performance through automated SMS	Basic mobile phones	CommCare	Africa	MCH and general health	Alerts & Reminders	x			x					x			x		x	x			x
Diero	2006	A computer-based medical record system and personal digital assistants to assess and follow patients with respiratory tract infections visiting a rural Kenyan health centre	Personalized Digital Assistant (PDA)	Mosoriot Medical Record System	Africa	Accute respiratory infections	Health Data Collection - Decision Support	x				x	x			x				x	x				
Dwolatzky	2006	Linking the global positioning system (GPS) to a personal digital assistant (PDA) to support tuberculosis control in South Africa: a pilot study	Personalized Digital Assistant (PDA)	Short Message Service (SMS)	Africa	HIV/AIDS, TB	Mapping		x			x				x					x				
Florez-Arango	2011	Performance factors of mobile rich media job aids for community health workers	Video-capable mobile phones	Windows Mobile Guideview	Central/ South America	Childhood illness, trauma, non-trauma	Decision Support			x		x	x			x				x				x	
Gisore	2012	Community based weighing of newborns and use of mobile phones by village elders in rural settings in Kenya: a decentralised approach to health care provision	Mobile phone & weighing scale	Short Message Service (SMS)	Africa	MCH	Health Data Collection		x			x		x	x	x				x					

Hoffman	2010	Mobile Direct Observation Treatment for tuberculosis patients: a technical feasibility pilot using mobile phones in Nairobi, Kenya.	Video-capable mobile phones	Short Message Service (SMS), Video	Africa	TB	Health Data Collection - Direct Observation	x			x	x		x		x				x			x		
Jones	2012	"Even if You Know Everything You Can Forget": Health Worker Perceptions of Mobile Phone Text-Messaging to Improve Malaria Case-Management in Kenya	Basic mobile phones	Short Message Service (SMS)	Africa	Malaria	Alerts & Reminders	x			x		x		x	x				x		x		x	
Lee	2011	Midwives' cell phone use and health knowledge in rural communities	Mobile phones equipped with GRPS technology	Short Message Service (SMS)	Asia	MCH, Sexual & reproductive health	Health Data Collection - Person-to-Person Communication		x			x	x		x	x				x			x	x	
LeMay	2012	Reaching Remote Health Workers in Malawi: Baseline Assessment of a Pilot mHealth Intervention	Basic mobile phones	FrontlineSMS	Africa	Sexual & reproductive health, HIV/AIDS	Information on Demand			x	x	x			x	x				x	x				
Mahmud	2010	A text message-based intervention to bridge the healthcare communication gap in the rural developing world	Basic mobile phones	FrontlineSMS Medic	Africa	HIV/AIDS, TB	Alerts & Reminders - Information on Demand - Health Data Collection - Decision Support - Pharmacy Management	x				x		x		x					x	x	x		
Mhila	2009	Using Mobile Applications for Community-based Social Support for Chronic Patients	Basic mobile phones	CommCare	Africa	HIV/AIDS	Health Data Collection - Electronic Medical Record		x		x					x				x					
Rajput	2012	Evaluation of an Android-based mHealth system for population surveillance in developing countries	Android mobile phone	Open Data Kit	Africa	HIV/AIDS	Health Data Collection	x			x	x				x				x	x				
Ramachandran	2010	Mobilizing health workers in rural India	Video-capable mobile phones	Short Message Service (SMS), Video	Asia	MCH	Multimedia Content		x		x	x			x	x				x				x	
Ramachandran	2010	Research and Reality: Using Mobile Messages to Promote Maternal Health in Rural India	Video-capable mobile phones	Short Message Service (SMS), Video	Asia	MCH	Multimedia Content		x		x				x	x				x					
Rotheram-Borus	2011	Project Masihambisane: a cluster randomised controlled trial with peer mentors to improve outcomes for pregnant mothers living with HIV	Basic mobile phones	Mobile Researcher	Africa	HIV/AIDS	Alerts & Reminders - Health Data Collection			x		x	x		x	x				x		x	x		
Svoronos	2010	CommCare: Automated Quality Improvement To Strengthen Community-Based Health The Need for Quality Improvement for CHWs	Basic mobile phones	CommCare	Africa	MCH	Alerts & Reminders - Decision Support - Electronic Medical Record	x			x		x			x			x	x				x	
Tomlinson	2009	The use of mobile phones as a data collection tool: a report from a household survey in South Africa	Basic mobile phones	Mobile Researcher	Africa	NA	Health Data Collection	x			x					x			x	x	x				
Zurovac	2011	The effect of mobile phone text-message reminders on Kenyan health workers' adherence to malaria treatment guidelines: a cluster randomised trial	Basic mobile phones	Short Message Service (SMS)	Africa	Malaria	Alerts & Reminders			x		x	x			x				x					
